# A Spatial Analysis of Suicide Displacement at a High-Risk Cliff-Based Location Following Installation of a Means Restriction Initiative

**DOI:** 10.1007/s11121-023-01504-6

**Published:** 2023-02-17

**Authors:** Michelle Torok, Jason Passioura, Paul Konings, Quincy Wong, Jiahui Qian, Mark E. Larsen

**Affiliations:** 1 Black Dog Institute, University of New South Wales, Sydney, NSW Australia; 2 National Centre for Geographic Resources & Analysis in Primary Health Care, Research School of Population Health, Australian National University, Canberra, Australia; 3 Centre for Mental Health Research, Research School of Population Health, The Australian National University, Canberra, Australia

**Keywords:** Suicide, Hotspots, Jumping, Spatial analysis

## Abstract

Means restriction interventions are recognised as highly effective for the deterrence of suicide attempts by jumping. While such interventions can lead to significant reductions in suicide, it is unclear whether these reductions represent a displacement effect, whereby individuals are instead choosing to attempt suicide at other nearby locations which offer the same means. The potential displacement of suicides as an unintended consequence of means restriction has been relatively unexplored to date. The only studies exploring displacement effects have focused on bridges, which are relatively easily contained sites; no studies have yet explored displacement effects at cliff-based high risk suicide locations (hotspots). Using Australian coronial data for the period of 2006–2019, we undertook joinpoint and kernel density analysis of suicides by jumping at a well-known cliff-based hotspot in Sydney, Australia, to determine whether there was evidence of displacement to local and broader surrounding cliffs following the installation of a multi-component harm minimization intervention (the Gap Park Masterplan). While slight decreases were noted in the immediate area subject to the structural intervention in the post-implementation period, alongside slight increases in the surrounding cliffs, there was no evidence for statistically significant changes. While kernel density analyses did not identify the emergence of any new hotspot locations in the post-implementation period, three existing hotspot sites of concern were found in our total area of interest, with greater than expected growth in the density of one of the hotspots. While we found no persuasive evidence of displacement, ongoing monitoring of the cliff-based location where the structural interventions were implemented is needed to ensure the ongoing safety of the area.

## Introduction

Preventing suicides at frequently used locations, or hotspots, often involves implementing structural means restriction interventions (e.g. installing barriers, fencing, or safety nets) (Yip et al., 2012). These interventions are designed to make it difficult to access high-risk jumping sites, and increasingly, signs, crisis phones, and closed circuit cameras are being implemented alongside structural barriers to also promote help-seeking and increase the likelihood of intervention. Several review studies have identified means restriction as one of the most effective suicide prevention strategies (Pirkis et al., 2013, 2015; Yip et al., 2012; Zalsman et al., 2016) based on evidence that such efforts can lead to both immediate and sustained reductions in both means-specific and population-level suicide rates (e.g. Beautrais, 2001; Berman et al., 2022). As structural means restriction interventions are arguably the most effective response to managing suicide hotspots, the ongoing monitoring and evaluation of these initiatives is vital to identifying and planning responses to address changes in their effectiveness. Evaluations of the efficacy of structural interventions have largely relied on examining change in the numbers or rates of suicide deaths at hotspots, comparing pre- and post-implementation periods (Pirkis et al., 2013). While this evaluation methodology is appropriate given it is difficult to test the efficacy of ‘real world’ structural interventions in controlled experimental trials, these trend analyses have primarily focused on demonstrating effectiveness only at the immediate implementation site (e.g. Beautrais, 2001; Lester, 2005; Ross et al., 2020). There has been a limited focus in such evaluation studies of whether there has been *displacement* of the hotspot to a new location within a reasonable proximity to the original hotspot. With method substitution being uncommon (Hawton, 2007; Yip et al., 2012), there is a risk that structural interventions at jumping sites may result in suicidal individuals choosing other close-proximity, accessible sites, which offer the same means. While potential jumping locations can include bridges, tall buildings, and cliffs – the latter is of particular interest. Cliffs, unlike bridges, may offer multiple jumping points that can be accessed with relative ease, and in close proximity, during periods of crisis, making them highly liable to displacement.

**Fig. 1:**
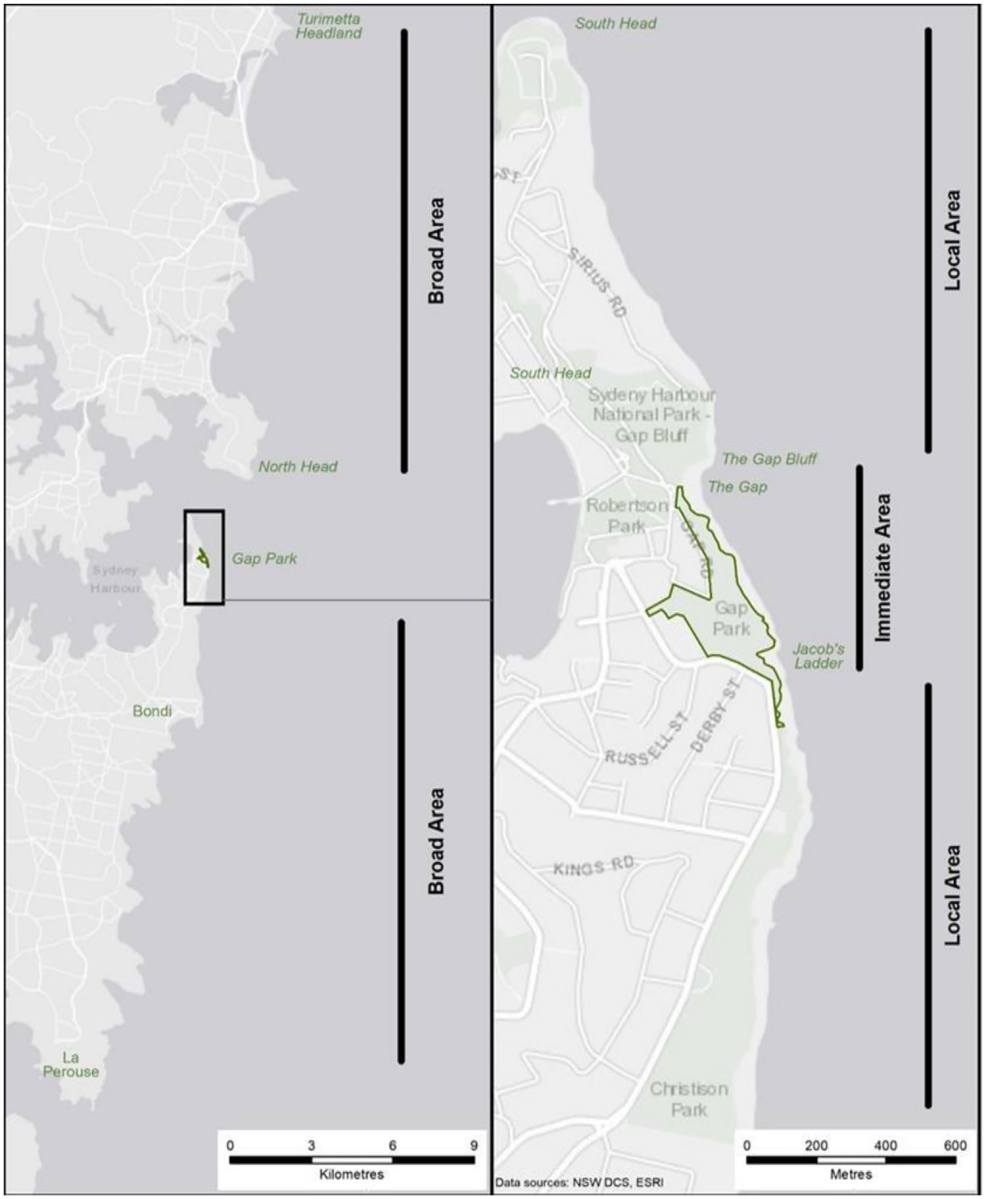
Map of geographic areas of interest for displacement

The potential displacement of suicides, and subsequent formation of new suicide hotspots, as an unintended consequence of means restriction has been relatively unexplored to date. The only studies exploring displacement effects have focused on bridges, with no evidence to support displacement to other bridges following barrier installation, even when two or more bridges are in close walking proximity to each other (Bennewith et al., 2007; Berman et al., 2022; Daigle, 2005; Law et al., 2014; Pelletier, 2007; Perron et al., 2013). No studies have yet examined displacement effects at high-risk cliff-based locations where a hotspot has been indicated. If displacement is occurring at cliffs, it raises questions about the effectiveness of means restriction efforts at these less easily contained locations. One of the most widely recognised coastal locations for jumping suicides is Gap Park, Sydney (Lockley et al., 2014). From 2010 to 2011, the Woollahra Municipal Council, in collaboration with several partners, established a structural intervention referred to as the Gap Park Self-Harm Minimisation Masterplan (Woollahra Municipal Council, 2008; referred to hereafter as the Masterplan). This Masterplan involved multiple strategies: the construction of an inwardly curved fence along the cliff edge at the main access point, installing help-seeking signage and phones that linked directly to a crisis service (Lifeline), and installing cameras. The study area, including Gap Park Masterplan, is shown in Fig. 1; however, there are stretches of coastline that extend north and south of Gap Park which offer similar opportunities for jumping, and which were not subject to the Masterplan initiatives.

Though the Masterplan has been in effect for a decade now, only two studies have evaluated the effectiveness of this initiative (Lockley et al., 2014; Ross et al., 2020). The most recent evaluation found that before the Masterplan was installed (2000–2010), suicide deaths were increasing among females and then decreased following its implementation (2011–2016), while male suicides followed a non-significant upward trend over the total study period (Ross et al., 2020). An earlier evaluation (Lockley et al., 2014) found a non-significant downward trend in jumping incidents from 2006 to 2012 (this included an assessment of the 12 months post the Masterplan’s implementation). No evaluations have yet spatially examined whether there are changes in suicide trends in the surrounding areas, nor whether new hotspots have emerged in the coastline surrounding the Masterplan area since its implementation. Examining displacement may provide new insights into the effectiveness of structural interventions at cliff-based jumping sites. As such, this paper aims to compare the pre- and post-Masterplan periods to identify if there is evidence of displacement of suicides from areas subject to means restriction activities in the immediate Gap Park Masterplan area to local and broader surrounding cliffs.

## Methods

This study presents a retrospective analysis of coronial records. Unit level (individual) data were acquired for registered deaths in Australia between 2006 and 2019 (inclusive) from the National Coronial Information System (NCIS). Data were included if the NCIS had coded the underlying cause of death as intentional self-harm (suicide) by jumping from a high place based on the International Classification of Diseases and Related Health Problems, 10th revision (World Health Organization, 2007), Australian Modification code X80. Records were allocated to either the ‘pre-Masterplan’ period (2006 to 2011, inclusive) or the ‘post-Masterplan’ period (2012 to 2019, inclusive) based on year of death.

For each suicide death in the defined period, the NCIS provided the additional following attributes:

- Incident location address, including either the street address (residential, business, etc.) or descriptor of the location (e.g. name of bridge, park, and lookout), as well as the suburb, postcode, and state/territory.
- Demographics (sex, age)
- Descriptor fields relating to the death, such as cause (e.g. ‘fall from height’), mechanism (e.g. ‘falling/stumbling/jumping/pushed from a height’) and object (e.g. ‘cliff’).
- Locational descriptor (e.g. ‘base of precipice, cliff’)

### Geocoding

As a first step, all NCIS records in the study period were subject to a standardised two-phase geocoding process to confirm that the suicide deaths occurred in areas of interest relevant to this study (Torok et al., 2021). If an NCIS address string could not be matched to a known address within the Australian Geocoded National Address File, it was manually geocoded using other address descriptors (e.g. ‘the gap proper’, ‘North head’, ‘South Head’) and online resources such as Google Maps.

After completing this initial geocoding phase, all NCIS records within proximity to the study area were subject to a rigorous assessment of the descriptors and any available case notes (police reports, coronial reports) to confirm that the death was the result of jumping and to allocate the most precise latitude and longitude coordinates for the incident location. Only records where a location could be geocoded within a precision of 500 m were included in the final analyses. Ten records were excluded from the final dataset during the geocoding phase as they could not be attributed to one of the three core areas of interest (immediate, local, broader) with this level of spatial precision.

All eligible records were then allocated to one of the following areas of interest for assessment of displacement (Fig. 1):

- **Immediate area**: includes the area from the southernmost point of entry to Jacob’s Ladder, northwards to Gap Bluff, covering the Masterplan area.
- **Local area:** includes the coastline from the tip of South Head, southwards to Clarke Reserve in Vaucluse (approximately 3 km north and south of the ‘immediate area’). This was chosen to assess close-proximity displacement as there is a continuous walking path connecting this area with Gap Park, and feasibly enables access to jumping points.
- **Broader area**: includes the coastline northwards to Turimetta Headland and southwards to La Perouse, representing distances approximately 18 km north and south of the ‘immediate area’ boundary.

To contextualise trends in suicide deaths by jumping from a height and assess for the possibility of methods substitution, we additionally examined all suicide deaths (by any means, i.e. ICD-10-AM codes X60-X84 [intentional self-harm], Y87.0 [sequelae of intentional self-harm]) that occurred in the Sydney area (covering statistical area level 4 codes: 115–128; Australian Bureau of Statistics, 2021) from 2006 to 2019. These data were extracted from NCIS records.

### Ethics

Ethics approval to access and use NCIS data was obtained from the Department of Justice and Community Safety Human Research Ethics Committee (JHREC) (reference number: CF/19/30711; M0422).

### Statistical Analysis

Our analytic approach focuses on identifying evidence of displacement following the installation of several structural self-harm minimisation strategies as part of the Gap Park Masterplan initiative. If there was a displacement effect, we would expect to see a significant increase in jumping suicides in the local or broader areas and a significant decrease in deaths in the Masterplan (Gap Park) area following its implementation. In addition to displacement, we also examined potential method substitution, examining all suicide deaths in the Sydney area. If substitution were present, we would expect to see a reduction in jumping deaths, offset by a parallel increase in the use of other methods. Joinpoint regression analysis was used to test changes in trends in numbers of suicides for 2006 to 2019 by area boundary (immediate, local, broader, total jumping, Sydney area – all suicide deaths) (Kim et al., 2000). By using suicide mortality counts as inputs, this method identifies the best-fitting points where a statistically significant trend change occurred. It calculates the annual percentage change (APC) in suicide counts between trend-change point, with 95% confidence intervals (95% CI). When there are no join points (i.e. no changes in trend), the APC is constant. The two periods of 2006–2011 and 2012–2019 were specified as Average Annual Percent Change periods in all models, to obtain a summary measure of the regression trends over these fixed time intervals. Time trends were presented as graphs, and for these, the annual suicide counts, and modelled regression data were binned (summed) into 2-year categories to obfuscate small numbers. Even with this approach, there remained one data point with fewer than four deaths in the local area, and this point was raised to *n* = 4 (in the graphs only) to be consistent with ethics requirements. Additionally, chi-square goodness-of-fit tests were conducted to determine whether the distribution of suicides by geographic area in the post-Masterplan period followed the same distribution as per the pre-Masterplan period. The hypothesised proportion of suicides that would be expected per geographic region during the post-Masterplan period was calculated based on the distribution in the pre-Masterplan period. The expected distribution of suicides in the post-Masterplan period are reported alongside actual (observed) counts (expected counts were rounded to the nearest whole number). If the omnibus chi-square analysis returned a significant result, standardised residuals (presented as *z*-scores) were calculated comparing the expected and observed counts per site to identify where the significant differences were. Where significance testing was appropriate, *p* values < .050 were considered significant. The software packages of SPSS Statistics 25.0 (IBM Corporation, 2017) and Joinpoint Regression Program version 4.9.0.0 (Statistical Methodology and Applications Branch, 2020) were used to conduct these analyses.

Kernel density analysis was conducted, which is a non-parametric, geoprocessing method principally used to create visualisations of point data that accounts for the location of features (e.g. destinations) relative to each other. The kernel function is based on the quartic kernel function described by Silverman (Silverman, 1986). A continuous surface grid is defined over the region, and the density at each surface grid cell is calculated by adding the values of all the proximal kernel surfaces where they overlay the raster cell centre. Locations with multiple proximal incident points received a greater weighting/higher score, while locations with increasing distance from the incident point receive a negligible weighting/score, reaching zero at the search radius distance from the point. The kernel density analyses visually depict where suicide incidents are sparsely distributed (dispersed) and where they are more concentrated (thus indicating hotspots), across the two time periods. A smoothing function (bivariate Gaussian distribution) adds the estimates of overlapping kernels for each cell (Guagliardo, 2004). Kernel density visualisations were created using a search radius of 300 m, which is appropriate for delineating potentially proximal feature densities on the narrow stretch of Eastern Sydney coastline included in this study. To help obfuscate the output (to prevent disclosure of individual incident locations), focus attention to the relatively narrow strip of cliffs, and provide a more realistic continuous surface of relative suicide prevalence, kernel density output was clipped to a 60 m buffer of the coastline. The distribution of suicides was weighted to account for the non-equivalence of the two time periods. Suicide incident data was analysed with ESRI ArcMap 10.8.1 (ESRI, 2015) using the kernel density geoprocessing function.

## Results

### Sample Characteristics

In 2006 to 2019, a total of 221 suicide deaths were confirmed as having occurred by jumping from a height within the immediate, local, and broader areas described in this study (‘jumping deaths’). The mean age at time of death was 42.2 years (SD: 16.3, range: 15–84 years), and 64.3% were male (*n* = 142). These characteristics were similar across the pre- and post-Masterplan periods (% male: 59% v 68%, *p* = .14; mean age: 43.5 v 41.1, *p* = 0.28).

Fifteen (6.8%) of the jumping deaths were by individuals who resided in the local postcode areas surrounding our immediate and local areas (2030, 2029, and 2026) at the time of death, showing that the majority of jumping deaths were by visitors to this area. Among all 221 persons who died by jumping from a height, the mean distance travelled from residential address to the final jumping location was 17.8 km (95% CI: 13.8–21.8 km), after replacing lowest and top 5% outlier data with upper and lower mean values. This means that the majority of the deceased resided in the Sydney area but were not local to the Gap Park area.

### Pre- and Post-Masterplan Trends, by Area of Interest

#### Displacement

**Fig. 2:**
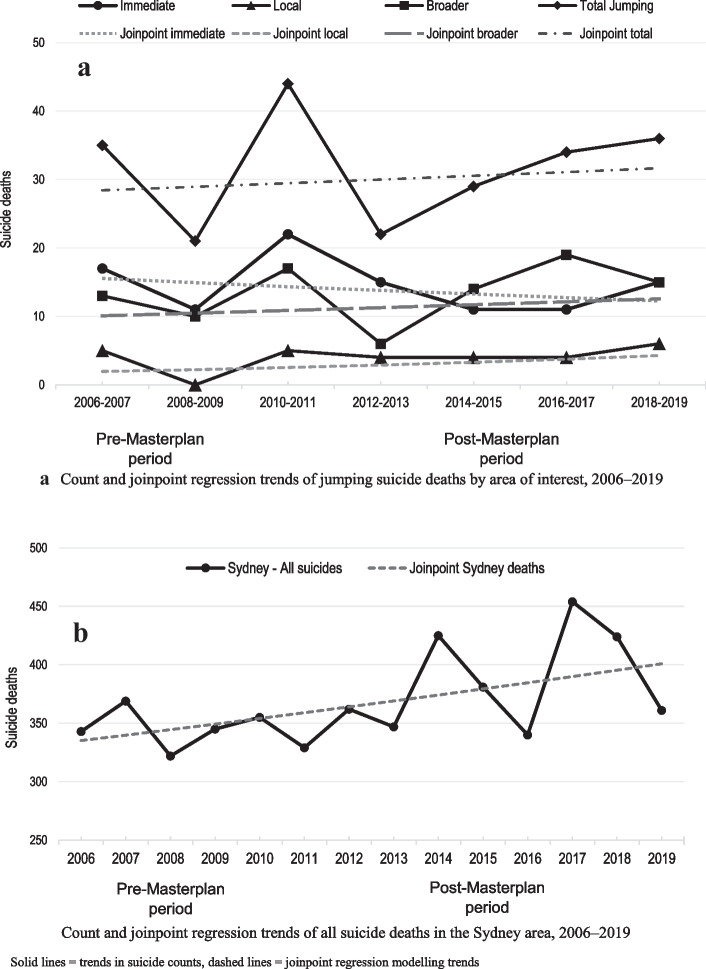
**a** Count and joinpoint regression trends of jumping suicide deaths by area of interest, 2006–2019. **b** Count and joinpoint regression trends of all suicide deaths in the Sydney area, 2006–2019

No join points were identified in any of the regression models, indicating that there were no significant trend changes in any areas in the time period of interest following the installation of the Masterplan (Table 1, Fig. 2a, b). As shown in Table 1, the there was a slight, non-significant downwards trend in the number of jumping suicide deaths in the immediate area (2% decrease) and a slight, non-significant, upward trend in the local area (APC = 6.8%) and broader area (APC = 1.9%).

**Table 1:** Characteristics of join point regression for each area of interest, 2006–2019

	**Time period: 2006–2019**				
*Area of interest*	*# Join points identified*	*Join point time periods*	*APC%*	*95% CI*	*p value*
Immediate area	0	2006–2019	−1.95%	−6.9, 3.3	0.140
Local area	0	2006–2019	6.81%	−4.6, 19.5	0.226
Broader area	0	2006–2019	1.85%	−7.4, 12.1	0.683
Total jumping deaths	0	2006–2019	0.90%	−3.9, 5.9	0.695
All suicide deaths, Sydney area	0	2006–2019	1.39%	0.1, 2.7	0.037

*APC* annual percentage change, *95% CI* 95% confidence interval

#### Method Substitution

There was no evidence of substitution from jumping deaths to other means. There was a small, but non-significant, upward trend in all jumping deaths in our areas of interest (aggregated) of 0.9% per annum (*p* = 0.695), and a slight, significant upward growth trend in all suicide deaths in the Sydney area for the same period (APC = 1.4%, *p* = 0.037) (Table 1).

### Expected Versus Observed Spatial Patterns

Chi-square testing showed there was no significant overall difference in the distribution of suicides across the geographic or hotspot areas across the pre- and post-Masterplan periods (Table 2). Though not significant, the number of suicides in the immediate area (i.e. the area covered by the Masterplan) was lower than expected in the post-Masterplan period and higher than expected in the local and broader areas (see Table 2).

**Table 2:** Actual versus expected number of suicides by geographical area by time period

	**Pre-Masterplan period** **(2006–2011)**		**Post-Masterplan period** **(2012–2019)**		
	*Actual suicide count*	*Expected suicide count*	*Actual suicide count*	*Expected suicide count*	*Significance*
**Geographic areas**					
Immediate	50	46	52	55	χ^2^(1) = 1.10, *p* = .18
Local	10	11	15	13	χ^2^(1) = 0.31, *p* = .37
Broader	40	42	54	51	χ^2^(1) = 0.48, *p* = .29
**Hotspot sites**					
The Gap	41	36	34	38	χ^2^(1) = 2.51, *p* = .08
Jacob’s Ladder	9	13	18	13	χ^2^(1) = 3.29, *p* = .05
North Head	12	12	13	12	χ^2^(1) = 0.01, *p* = .55

**Fig. 3:**
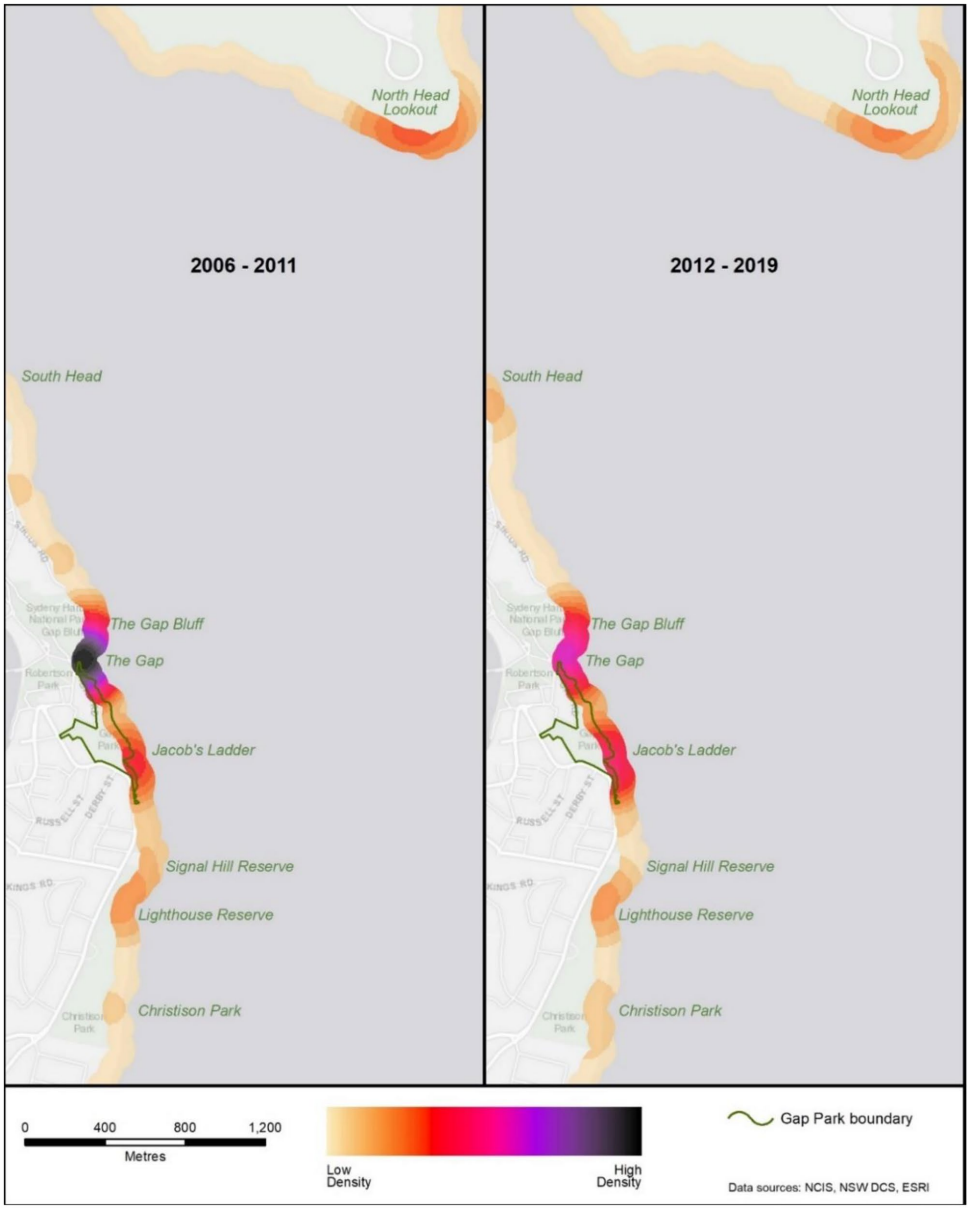
Kernel densities of suicide hotpots in the pre- and post-Masterplan periods

In addition, kernel density analyses were conducted to identify suicide hotspots in the total geographic area of interest. Three hotspots were found, located at the Gap (immediate area), Jacob’s Ladder Lookout (immediate area), and North Head Lookout (broader area) (Fig. 3). Compared to the expected distribution of suicides, there was a greater number of actual suicides during the post-Masterplan period at Jacob’s Ladder than expected (*z* = 1.80, *p* = .05) but not for the Gap (*z* = 1.60, *p* = .08) or North Head (*z* = 0.10, *p* = .55) (Table 2). As Fig. 3 shows, all three hotspots existed in the pre-Masterplan period and remained hotspots in the post-Masterplan period.

## Discussion

In this study, we sought to determine if there was evidence for the displacement of suicides by jumping to other nearby cliff-based locations (i.e. those with fewer impediments to access) following the installation of the Gap Park Masterplan, which, in part, was designed to prevent suicides by restricting access to jumping points. To our knowledge, this is the first study to examine displacement at a cliff-based hotspot location, and accordingly, it adds novel insights to current understandings of the effectiveness of structural (means restriction) interventions. In the 8 years following the implementation of the Masterplan initiative, we found no persuasive evidence of displacement in jumping deaths in any of the local or broader cliffs adjacent to the Gap Park area. This finding is not entirely surprising, given we would only expect to be able to detect displacement if there was a significant change (decrease) in suicide deaths at the site where the means restriction intervention was implemented to allow for a change (increase) at other jumping locations. As per prior studies (e.g. Lockley et al., 2014; Ross et al., 2020), we found that there have been no significant reductions in jumping suicide deaths following the implementation of the Masterplan initiative. There was also no evidence to support method substitution, as both jumping deaths *and* suicide deaths by all other methods increased during the study period. The net effect of this slight increase in suicides should be monitored and further analysed to identify factors driving these trends, to inform future directions for prevention.

While our findings do not support displacement from the Masterplan area to surrounding local or broader coastlines, there was some evidence of density changes to existing suicide hotspots in Gap Park which may suggest immediate displacement. That is, the actual (observed) number of suicide deaths at Jacob’s Ladder Lookout was greater than expected in the post-Masterplan period, alongside slightly fewer than expected deaths at the Gap proper. The close proximity of these two sites, which are approximately 500 m from each other, may not only indicate an unwillingness to substitute methods, but a willingness of those in acute suicidal distress to find sites that offer the same means with fewer impediments to access and/or visibility. Further research is needed to understand the motivations for site choice among individuals considering suicide, or who have attempted suicide, at this hotspot location. Such knowledge will be vital to informing the optimisation of existing means restriction interventions and enhancing their efficacy.

Our findings suggest that effective means restriction at cliff-based hotspots is challenging. Unlike bridges, the natural geography of cliff-based sites means that there are likely to be multiple access points for jumping over extended distances. Where we have seen structural means interventions (e.g. safety fences or barriers) used remarkably effectively at bridge-based suicide hotspots, reducing suicide deaths between 50% (Bennewith et al., 2007; Law et al., 2014) and 100% (Berman et al., 2022; Pelletier, 2007; Perron et al., 2013) within 5 years of implementation, there is currently no similar evidence for suicide deaths at cliff-based sites. The general lack of studies evaluating means restriction interventions at cliff-based sites means that current understandings of what strategies would work at such challenging locations is very limited. However, in the context of the bridge-based literature, one question raised from our current study is whether the current fences installed as part of the Masterplan are of appropriate height to create sufficient difficulty in reducing site access. For example, the inwardly curving fences where the Gap and Jacob’s Ladder hotspots are located are approximately 1.3 m high. A recent study of suicide prevention measures at bridges and buildings identified that barriers of at least 2.3 m in height are needed to effectively deter suicide attempts by jumping (Hemmer et al., 2017). At this height, the fence becomes ‘unclimbable’, and this could allow a suicidal crisis to pass without fatal effect (Yip et al., 2012). While there have been some additional upgrades to the Gap Park Masterplan since 2011, including additional investment in more closed circuit television cameras (cctv), and a virtual fence was commissioned in 2013 that alerts police when human movement is detected on the cliffs (Ly, 2016), there have been no modifications made to the existing physical fencing. The absence of clear evidence for prevention effects, despite ongoing upgrades to the Masterplan, not only raises questions about the effectiveness of non-structural, public safety measures (cctv, lighting, crisis phones) at suicide hotspots, but suggests that high (unclimbable) physical fencing is perhaps *the* most crucial component of the effectiveness of means restriction at jumping locations.

### Strengths and Limitations

A major strength of this study is geo-accuracy of our data – and the subsequent validity of our findings. Approximately 90% of the incident cases were able to be coded within 200 m or less of a jumping point; this specificity was achieved through a combined manual and auto-geocoding process and is far more accurate than what would be possible when using the geocoordinates provided by the data custodian only (Torok et al., 2021). Our prior research shows that approximately 50% of suicide deaths which occur at feature-based locations (e.g. cliffs, bridges) need to have their geocoordinates revised by more than 1000 m (Torok et al., 2021). However, despite our rigorous efforts to identify all relevant cases in the geographical area of interest and assign them to the most accurate location, it is possible that errors were made, which risks under-representing the size and accuracy of the problem. Also, very small numbers of people die by jumping from a height in any single ‘hotspot’ location. The data sparseness precluded us from using inferential spatial analyses, such as Poisson-based modelling. Instead, we were only able to confidently conduct kernel density analysis, which is a descriptive, non-parametric way to estimate the probability density of suicide deaths in the geographic area of interest which means we cannot comment on the statistical significance of hotspots. Displacement effects may have also been undetected due to this data sparseness, and future studies could benefit from including data on suicide attempts collected via police or ambulance to increase power. Finally, we caution that the findings of this study may not be generalizable to other cliff-based jumping locations given that the fences implemented as part of the Masterplan initiative were lower than what is recognised as an effective deterrent height (Hemmer et al., 2017). Further exploration of potential displacement at cliff-based sites where full height fences have been installed, creating optimal conditions for prevention, will contribute an important depth of understanding to the issue of means restriction effectiveness.

## Conclusion

To the best of our knowledge, this paper presents the first examination of whether means restriction interventions result in displacement of suicides at high-risk cliff-based jumping locations. It advances current understandings of ‘effectiveness’ in the means restriction literature, as evidence for displacement would suggest that structural initiatives may either lose effectiveness over time or have unintended consequences. There was no evidence of potential displacement to the nearby local or broader surrounding coastline areas; however, displacement likely could not be identified given the Masterplan at Gap Park is, at present, not effectively reducing suicides. Studying displacement effects at cliff-based hotspots where means restriction interventions have been effective will be critical to advancing knowledge of this phenomenon, and of what works to prevent suicide at cliff sites. We did, however, find some evidence of growth in alternate jumping locations within Gap Park itself, which will require monitoring to intervene effectively to improve public safety. While it is unclear if there is a cost-benefit in extending means restriction efforts beyond the Gap Park for now, consideration should be given to further physically obstructing access within the immediate area if suicide deaths are to be prevented.

## Data Availability

The data underlying this article cannot be shared publicly due as it is highly sensitive in nature and is owned by the Department of Justice and Community Safety. As per our ethics agreement, we are unable to distribute the data further without express agreement from the National Coronial Information System.
